# The new criteria for a COVID19 patient for the clinical practice to determine the need for an early therapeutic regimen and to decrease mortality

**DOI:** 10.12669/pjms.37.5.3630

**Published:** 2021

**Authors:** Mulazim Hussain Bukhari, Shahzadi Zain, Mobeen Syed

**Affiliations:** 1 Mulazim Hussain Bukhari, Principal Azad Jammu Kashmir Medical College, Muzaffarabad, Azad Jammu and Kashmir, Pakistan; 2 Shahzadi Zain, Schulich School of Medicine and Dentistry, Western University, Canada; 3 Mobeen Syed, DRBEEN.COM Personalized Medical Education, California, USA

**Keywords:** COVID19, Cytokine Storm, Pro-inflammatory status, Systemic cell death, Multi-organ tissue damage, Pre-renal electrolyte imbalance, SARC-CoV-2

## Abstract

A new predictive criterion is being proposed for the determination of cytokine storm (CS) in COVID-19 (COVID-CS). It is comprised of results of laboratory that associate the pro-inflammatory status, systemic cell death, multi-organ tissue damage, and pre-renal electrolyte imbalance. The data identifies the patients’ stay in hospitals and their mortality with the relevance of hyper-inflammation and tissue damage during the CS. The criteria can be readily used in clinical practice to determine the need for an early therapeutic regimen, block the hyper-immune response and possibly decrease mortality. It helps to understand the nature of the virus by following a specific criterion to predict the disease. The SARS-CoV-2 tells us in few days what nature has decided for the patient i.e., recovery, death or permanent disability.

## INTRODUCTION

The current COVID19 pandemic emerged from China in December 2019, spread throughout the world, and now we are grappling with its second wave. The second and third waves of COVID-19 are on the rise all over the world. The doctors and scientists from all over the world including Pakistan are playing their role in saving the humanity.^1^,^2^

The proposed criteria can be readily used in clinical practice to determine the need for an early therapeutic intervention, block the Cytokines Storm (CS) and possibly reduce mortality. We all know that people who go into CS are the ones who are most at risk and have the high mortality rate. There is a need to assess this early to save the patient in this condition.^3^-^5^

Previously, there were few criteria to assess the condition of patients suffering from COVID-19 i.e., haemophagocytic lymphohistiocytosis (HLH) and macrophage activation syndrome (MAS) which cannot validate the association of CK storm and survival of the patients. HScore was used to see the survival of patients but there were so many flaws to predict the condition of the patients.^3^,^4^

To select a new criterion, a study which was conducted from 3^rd^ of March to 3^rd^ of April 2020, collected 9 labs values including CBC, differential count, metabolic tests, inflammatory markers and IL. They believe that the cause of COVID-19-related cytokine storm (CS) may be due to the over activity of human immune system, which is called the hyperimmune response and consequently patients infected with COVID-19 may lead to multi organ damage, including the lungs.

Earlier it was thought that there could be two causes of inflammation and tissue destruction, but we found there are four causes which will be mentioned later. This was revealed by a study conducted on 513 people whose COVID-19 test was positive on PCR and there were white, ground-glass opacity on high-resolution CT of the lungs. It was analyzed by studying the laboratory results of the first seven days of hospitalization. A criterion was developed based on these findings. This criterion was also validated with a second cohort of 258 patients.

### The Cytokine Storm could not be predicted by previous criteria

These results were compared with the results of another study of 258 patients and then validated to develop the current standard. It was observed that the scoring system of the previous cytokine Storm was not effective and did not identify the COVID-19 cytokine storm (COVID-CS).^3^,^4^

### The Criteria of the day to predict the COVID-CS

Researchers found this new standard to be better for estimation of cytokines storm and proved to be very useful in determining the prognosis of the patients suffering from COVID19. In the study it was also found that prolonged hospital stays were also a sign of bad prognosis (Longer stay in hospital was also dangerous). As the SARS-CoV-2 attack progresses, there is systemic-inflammation, and due to hyper immune response, there is more tissue damage which puts the patient at higher risk.^3^,^4^,^6^,^7^

This new criterion can be easily used in clinical practice to predict patients’ prognosis. If all the doctors use this criterion then patients can be saved from death and further harm, and future of the patient can be predicted easily. The criteria can be readily used in clinical practice to determine the need for an early therapeutic intervention, curb the hyper-immune response and possibly decrease mortality. These patients were on oxygen. In addition, they were taking prednisolone in low doses, i.e., 3 mg / kg body weight and azithromycin. When the patient is taking steroids, the chances of infection increase, so antibiotics are necessary. They applied MAS and HLA criteria but both failed to predict the patients’ future. They saw that despite the treatment, a Cytokine storm broke out among these people. Hence, a newly devised consensus was based on (1) worsening respiratory status defined as increased oxygen supplementation required to maintain SpO_2_>93% and (2) elevation above threefold the upper normal level of at least two of the following markers: C reactive protein (CRP), ferritin, D-dimer, lactate dehydrogenase (LDH) and cardiac troponin. Patients in this group were retrospectively selected as control for the following statistical analyses. Two of the five clinical trials found that there were three times more than the normal levels of CRP, D dimmers, Serum ferritin, LDH and-Troponin I (These patients had also taken Tocilizumab).^8^

### How a doctor will know which patient will have a cytokine storm?

When the pre-existing criteria were applied to these patients and they were found to be only partially effective. When the LHA criteria was applied, it could only predict two patients to be in worrisome situation. When HScore criteria was applied, it could only identify three patients that would be in an alarming situation. When the MAS criteria was applied, it could only predict one patient that would be in a worrying situation. In summary all these could not be applicable to patients with COVID-19 to assess and foresee the cytokines storm.^8^

### New Criteria to assess the COVID-19 Cytokines storm

All the Patients were having the symptoms of COVID-19, but the RT-PCR does not have to be positive. The CT scan or X-ray showed ground glass patches were present in all of these patients. Serum ferritin was more than 1ng/ml. CRP was three time more than the normal levels i.e., above 4.5 mg / dl. One of the tests from each of these three clusters of the following laboratory groups, were significantly changed, i.e., either increased or decreased.^8^

### Deterioration in COVID19-CS (Table-II)

**Table-I:** Clustering of the Patients according to the Lab reports with Cytokine Storm (CK Storom).

Clustering (with Normal Value)	CK Storm	No CK storm	Significance (p-Value)
***Cluster 1***			
Serum albumin (2.9±0.6g/d)	<2.8	2.9±0.6	0.001
Lymphocytes (20%–40%)	<10.2	19±11	<0.001
Neutrophil Absolute Count			
1.8–7.8K/mm3	>11.4	5.8±3.6	0.004
***Cluster 2***			
AST (29-33U/L)	>60	50±80	0.04
ALT (5-40U/L)	>87	43±37	0.028
D-Dimers (>500 ng/ml)	>4930	2,396±7,851	>0.002
LDH (140-280U/L)	>416	305±153	<0.001
Troponin (0.045–0.1ng/mL)	>1.2	?0.1±0.38	<0.001
***Cluster 3***			
Anion Gap (6–16?mmol/L)	< 6.7	7.8±3.0	<0.001
Chloride (101–111mmol/L)	> 106	104±5	0.032
Potassium (3.5–5.2 mmol/L)	> 4.9mmol/L	4.07±0.51	0.019
BUN (10–20 ratio)	> 29 ratios	18.5±8	0.03

**Table-II:** Laboratory investigation of Cytokine storm in COVID-19.

Systemic Inflammation	Immunity	Tissue damage	Increased Pre-renal and renal damage
Increase ferritin	Innate Immunity	Increased ALT/AST (Liver damage)	Cl-, K+, Na+ Electrolyte imbalance
Increase CRP	Increase Neutrophils count	Increased D-Dimers & low Platelets (endothelial damage/ thrombosis/ coagulation)	Creatinine
Increase Triglycerides	Increase monocytes count	Increase Troponin I (Heart damage)	BUN
Decrease albumin	Adaptive Immunity	Increase LDH/ESR (cell death/tissue damage)	GFR
IL-6	Decrease Lymphocytes/NC	Urea (Kidney)	Urine Protein

There are four possible causes which result in COVID-CS during the attack of SARS-CoV-2 in humans, including Systemic inflammation, Immune system deregulation, Tissue damage of human system and Pre-renal and renal damage (pretension overload and renal damage) resulting in variation in many laboratory parameters.

### A- Systemic inflammation

When the SARS-CoV-2 attacks, and there is CS, there is inflammation throughout the body, resulting in in the rise of serum ferritin, CRP, Interleukin-6 (IL6), Triglycerides and, decrease in total albumin levels.

### B- Immune system deregulation

During this situation there is over activity of innate immune system with increased neutrophils, and monocytes. It also results in dysfunctional adaptive immune system resulting in decreased lymphocytes.

### C- Tissue damage of human system

During such situation, there is tissue damage throughout the body and markers such as liver enzymes (ALT and AST) level is increased. When there is endothelial damage, there is increase in LDH level indicating generalized cell death and high Troponin I showing damage of heart tissues.

### D- Pre-renal and renal damage (pretension overload and renal damage)

During COVID-CS, there is prerenal overload and renal damage and, in this case, the following markers may become abnormal.

1. Potassium (K^+^)(May be normal)
2. Calcium (Ca^++^)(May be normal)
3. Chloride (Cl^-^)(May be normal)
4. Blood Urea
5. Serum creatinine
6. Blood Urea Nitrogen (BUN)

## CONCLUSION

The new criteria facilitate the diagnosis of Cytokines storm during COVID-19 at an early stage, which could predict the longer hospitalization and poor prognosis.

### Authors’ Contributions:

**MHB,** is the main author, who conceived, designed the study, and edited the manuscript, is responsible for integrity of study.

**SZ,** revised the article and reviewed the literature related to this article.

**MS** Reviewed and gave the final approval of the manuscript.
